# A community pharmacy weight management programme: an evaluation of effectiveness

**DOI:** 10.1186/1471-2458-13-282

**Published:** 2013-03-27

**Authors:** David Morrison, Philip McLoone, Naomi Brosnahan, Louise McCombie, Andrea Smith, Janie Gordon

**Affiliations:** 1 West of Scotland Cancer Surveillance Unit, Public Health Research Group, Institute of Health and Wellbeing, University of Glasgow, Glasgow G12 8RZ, Scotland, UK; 2 Counterweight Ltd, c/o Brodies LLP, 15 Atholl Crescent, Edinburgh EH3 8HA, Scotland, UK; 3 NHS Fife, Pentland House, Lynebank Hospital, Halbeath Road, Dunfermline KL11 8JH, Scotland, UK

**Keywords:** Obesity, Weight management, Pharmacy, Counterweight, BMI

## Abstract

**Background:**

Community pharmacies may offer an accessible way of delivering weight-management programmes but there have been few trials that use clinically significant weight loss outcomes, objective measures of weight and follow-up to 12 months. We aimed to evaluate weight change among patients who used the Counterweight weight management programme delivered by community pharmacies.

**Methods:**

The Counterweight Programme was introduced into community pharmacies in Fife, Scotland in 2009 for patients with a BMI ≥ 30 kg/m^2^ or a BMI ≥ 2830 kg/m^2^ with a co-morbidity in localities in which Counterweight was not available at GP practices. The aim was to achieve an energy deficit of 500-600 kcal per day. Counterweight specialist dietitians delivered training, support and patient information materials to community pharmacies. Patient weight was measured by pharmacy staff at each weight management session. Weight data recorded at each weight management session were used to estimate weight change and attendance at 3, 6 and 12 months.

**Results:**

Between March 2009 and July 2012, 458 patients were enrolled by the community pharmacies. Three-quarters of patients were women, mean age was 54 (SD 7.4) years and mean BMI 36.1 (SD 5.9) kg/m^2^. Of 314 patients enrolled for at least 12 months, 32 (10.2% on an intention to treat basis) had achieved the target weight loss of ≥5%; this was 41.6% of those who attended at 12 months representing a mean weight loss of 4.1 kg. Using Last Observation Carried Forward, 15.9% achieved the target weight loss within 12 months of enrolling. There was no significant effect of sex, baseline BMI or age on weight loss.

**Conclusions:**

The Counterweight pharmacy programme has a similar effectiveness to other primary care based weight management programmes and should be considered as part of a range of services available to a community to manage overweight and obesity.

## Background

Obesity is an important risk factor for many chronic conditions, such as cardiovascular diseases, cancers and diabetes mellitus [1-3]. The worldwide prevalence of obesity has doubled since 1980 [4]. In the UK it has trebled in the past thirty years and further increases are predicted [5,6]. A range of approaches will be needed to halt the increase in obesity and to reduce its prevalence. These will include primary prevention by engineering public places to promote physical activity and encouraging the avoidance of unhealthy foods [7]. Effective treatment of overweight and established obesity, however, will also be needed [8]. Several models for providing obesity management in primary care have been reported, including the use of community pharmacies.

It is estimated that 95% of the population visit a community pharmacy during the year [9,10]. Because of this, pharmacies in Scotland have been encouraged to provide a variety of health related services - these have included health assessments for diabetes, cardiovascular diseases, asthma, and smoking cessation [9]. Pharmacies also offer over-the-counter weight loss products [11]. Where pharmacy has been used as one treatment arm in a randomised controlled trial comparing a range of weight reduction programmes, 14% of pharmacy participants lost at least 5% of their initial weight although there was no significant difference in their mean weights at 12 months [12]. A recent systematic review of the effectiveness and cost effectiveness of community pharmacy-based weight management identified 10 initiatives involving 582 pharmacies in 5 countries. The authors of the review found that community pharmacy weight management could produce modest weight loss at 12 months of between 1.1-4.1 kg, but concluded that there was insufficient evidence of effectiveness and cost effectiveness because of limitations in how the studies were carried out and reported [13]. For example, only 3 studies reported long-term (12 month) weight change; only one study employed evidence-based guidelines in its programme; and few reported any cost information. The authors of the review recommended that body weight should be objectively measured and that proportions of patients achieving clinically significant weight loss should be reported alongside mean weight loss to enable comparison of effectiveness from a clinical point of view [13]. The health benefits of clinically significant weight loss, defined as loss of ≥5% baseline weight, include reduced blood pressure, improved glycaemic control, reduction in risk of type 2 diabetes, improved lipid profiles, and reduced osteoarthritis-related disability [14].

Counterweight is a weight management programme that has been evaluated for use in routine National Health Service primary care [15,16]. A case series study showed that it achieved clinically significant long-term weight loss in 14% of all patients at 12 months [16]. The programme was introduced into community pharmacies in the Fife region of Scotland in 2009. Our aim was to evaluate the effectiveness of the Counterweight Programme delivered within community pharmacies, using a primary outcome of clinically significant weight change at 12 months.

## Methods

### Counterweight intervention

The Counterweight weight management programme was provided in the Fife region (population 365,000) as part of the Keep Well project. The Keep Well project encourages 40 to 64 year olds, who live in geographical areas which have been identified as having greatest need, to improve their health. The program targets those individuals at high risk of cardiovascular disease, and offers medical advice and support through enhanced Primary Care services. Between 2008 and 2010, 50 general practices in Fife were engaged in the delivery of the Keep Well project. Patients registered with participating practices who had a BMI ≥ 30 kg/m^2^ or a BMI ≥ 28 kg/m^2^ with a co-morbidity and who were assessed as being motivated to lose weight were referred to the Counterweight Programme.

Eighty community pharmacies were approached to determine interest in delivering the Counterweight Programme. Twenty three pharmacies expressed an interest, and 18 were invited because they were located in geographical areas where local general practices did not deliver Counterweight. Sixteen pharmacies subsequently agreed to deliver the programme and received training. Twelve of the participating pharmacies where situated in small urban settlements [17] with between 10,000 to 125,000 inhabitants. The remaining four were located in small towns of 3,000–10,000 people. Participating pharmacies were required to have a private consultation room and time to deliver the intervention. All pharmacies had extended opening hours and offered appointments in the evening and at weekends. Marsden High Capacity Portable Scales (Class III) and Seca Leicester Portable Height Measures were supplied by the NHS Fife Keep Well project to measure weight and height. Scales were calibrated on an annual basis [18]. Counterweight resources (training manuals, desk top flip charts and patient information booklets) were initially funded through the core Counterweight Scottish Government funding. Pharmacies were paid a single commitment fee of £100 for taking part, plus a payment per patient and payments for the provision of replacement staff while staff were trained . Between March 2009 and May 2010, the payment per patient was £54, which comprised £30 for patients attending 1-3 appointments and a further £24 for patients attending 4 or more appointments. From May 2010 to date these payments rose to £64 and £40, respectively.

Specialist dietitians competent in Counterweight Programme delivery conducted two four-hour training sessions and a further 3 hour session after 6 months to consolidate the initial training. Most trained staff were pharmacy assistants rather than pharmacists. It was agreed that pharmacy staff would not sell over-the-counter weight loss medications to patients enrolled in the programme. The specialist dietitians also provided mentoring to all pharmacies.

The Counterweight approach to weight management has been described in detail elsewhere [16]. In brief, pharmacy staff delivered patient education by discussing weight management, and communicating information on behaviour change strategies. The initial interventions involved a prescribed eating plan or a goal-setting approach. The aim was to achieve an energy deficit of 500-600 kcal/day. As patients progressed through the programme, emphasis was increasingly directed to weight loss maintenance and the prevention of weight regain. Patients were asked to commit to nine appointments in 12 months following the initial screening visit. This included six initial appointments (10–30 minutes each) with follow-up visits at 6, 9 and 12 months. The total time for one patient to be taken through the full programme was estimated at 130 minutes.

The data collected at each visit were recorded using paper based patient forms. Anonymised patient forms were collated centrally and entered into a bespoke Microsoft Access database. The data were checked for incomplete or inaccurate information. The central database was sent to an independent team at Glasgow University at set time points.

### Ethics

Formal ethical approval was unnecessary as this was an audit of a planned delivery of an existing intervention, no new or untested treatment was being offered, and there was no experimentation. No personally identifiable data were collected and written consent was not required.

### Data definitions

Baseline weight and height were collected when patients attended the first session of the weight loss programme. During the first appointment patients were asked whether they smoked and whether they had diabetes. Their response was recorded as a binary variable (yes/no). Smoking and diabetes status were recorded because they are relevant factors associated with weight, attendance and weight loss. Weight was measured at each subsequent visit. Weight change was evaluated at 3, 6 and 12 months. The weight measurements used in the evaluation were recorded in kilograms at dates closest to 3, 6 and 12 calendar months from enrolment (within time frames of 6–15 weeks, 15 weeks–9 months, and 9 – 18 months respectively). The primary outcome of the study was weight loss of at least 5% of baseline weight at 12 months.

### Statistical methods

Descriptive statistical methods were used to present change in weight at 3, 6 and 12 months. Values are presented as means and 95% confidence intervals (95% CI), if not indicated otherwise. We present weight change as absolute weight change and the percentage achieving at least 5% weight loss. The analyses were carried out separately for patients who attended at each time point, and for all patients assuming that participants for whom weight at follow-up was not available retained their baseline weight (baseline-observation-carried-forward BOCF), and assuming participants retained their last observed weight (last-observation-carried-forward LOCF). BOCF and LOCF were included to enable comparison with other studies, but they are biased methods of imputing missing data [19].

Kruskal-Wallis one way analysis of variance, the chi-square test for differences in proportions and logistic regression were used to examine the association of age, sex, and starting BMI with weight loss and attendance. Age and BMI were employed both as continuous and categorical variables. Age was categorised into 3 groups (<50, 50-59, and 60+ years). BMI was categorised as follows <30, 30- < 35, 35- < 40, and 40+ kg/m^2^. The conventional statistical significance threshold of 5% was used (p < 0.05). All analyses were conducted with STATA version 11 (StataCorp, CollegeStation, TX, USA).

## Results

Between March 2009 and July 2012, 458 patients were enrolled by 16 community pharmacies. The baseline characteristics of patients are shown in Table 1. Sex, age and BMI were not recorded for 2 (0.4%), 12 (2.6%) and 6 (1.3%) patients respectively. Three-quarters of patients were women, mean age was 54 years and mean BMI was 36.0 kg/m^2^. One-fifth of patients had a BMI of 40 kg/m^2^ or over. Smoking was reported among 14% of patients. Patients who smoked had a slightly lower mean BMI 34.3 kg/m^2^ (95% CI 33.0, 35.6) than those who reported that they did not smoke 36.6 kg/m^2^ (35.9, 37.3) (p = 0.005). One in ten patients reported diabetes. Patients with diabetes had a higher baseline BMI 39.1 kg/m^2^ (37.0, 41.2) than patients who did not have diabetes 35.8 kg/m^2^ (35.2, 36.4) (p = 0.002).

**Table 1:** Baseline characteristics of 458 Counterweight patients enrolled in community pharmacies

**Patients (n = 458)**			
**Baseline characteristics**		**% or mean**	**(n or SD)**
**Men**%		24.9	(114)
**Women**%		74.7	(342)
**Age** mean years (SD)		54.0	(7.4)
**Weight** mean kg (SD)		96.4	(18.3)
**Starting BMI** mean kg/m^2^ (SD)		36.0	(5.9)
**Starting BMI** kg/m^2^%	<30	9.8	(45)
	30-34	43.9	(201)
	35-39	23.8	(109)
	≥40	21.2	(97)
	not recorded	1.3	(6)
Smoking status%	smoker	14.4	(66)
	not recorded	18.8	(86)
Diabetes Status%	diabetic	11.6	(53)
	not recorded	15.7	(72)

Attendance and weight change at 3, 6 and 12 months are shown in Table 2. Of the 458 patients who started Counterweight within the enrolment period, progressively fewer had been in the programme long enough to be eligible to attend at later time points. Attendance declined over time from 56.0% at 3 months to 24.5% at 12 months. Of 314 patients enrolled for at least 12 months, 32 (10.2%) had achieved the target weight loss of ≥5%. This represents 41.6% of those who attended at 12 months.

**Table 2:** Weight loss among patients at 3, 6, and 12 months since starting programme

		**Time since starting programme**		
		** 3 months n = 430**	** 6 months n = 395**	** 12 months n = 314**
		% or mean (n or 95% CI*)	% or mean (n or 95% CI*)	% or mean (n or 95% CI*)
**Attendance**% (n)		56.0 (241)	33.7 (133)	24.5 (77)
**Weight loss** mean kg (95% CI*)	Attending patients	2.4 (2.02, 2.70)	3.5 (2.66, 4.25)	4.1 (2.83, 5.41)
	BOCF**	1.3 (1.10, 1.54)	1.2 (0.85, 1.58)	1.0 (0.64, 1.38)
	LOCF***	1.3 (1.10, 1.54)	1.6 (1.25, 1.89)	1.7 (1.31, 2.14)
**≥5% weight loss**% (95% CI*)	Attending patients	17.0 (12.5, 22.4)	34.6 (26.6, 43.3)	41.6 (30.4, 53.4)
	BOCF**	9.5 (6.9, 12.7)	11.6 (8.7, 15.2)	10.2 (7.1, 14.1)
	LOCF***	9.5 (6.9, 12.7)	13.9 (10.7, 17.7)	15.9 (12.1, 20.4)

*95% confidence interval.

**BOFC - baseline observation carried forward.

***LOFC - last observation carried forward.

**Figure 1:**
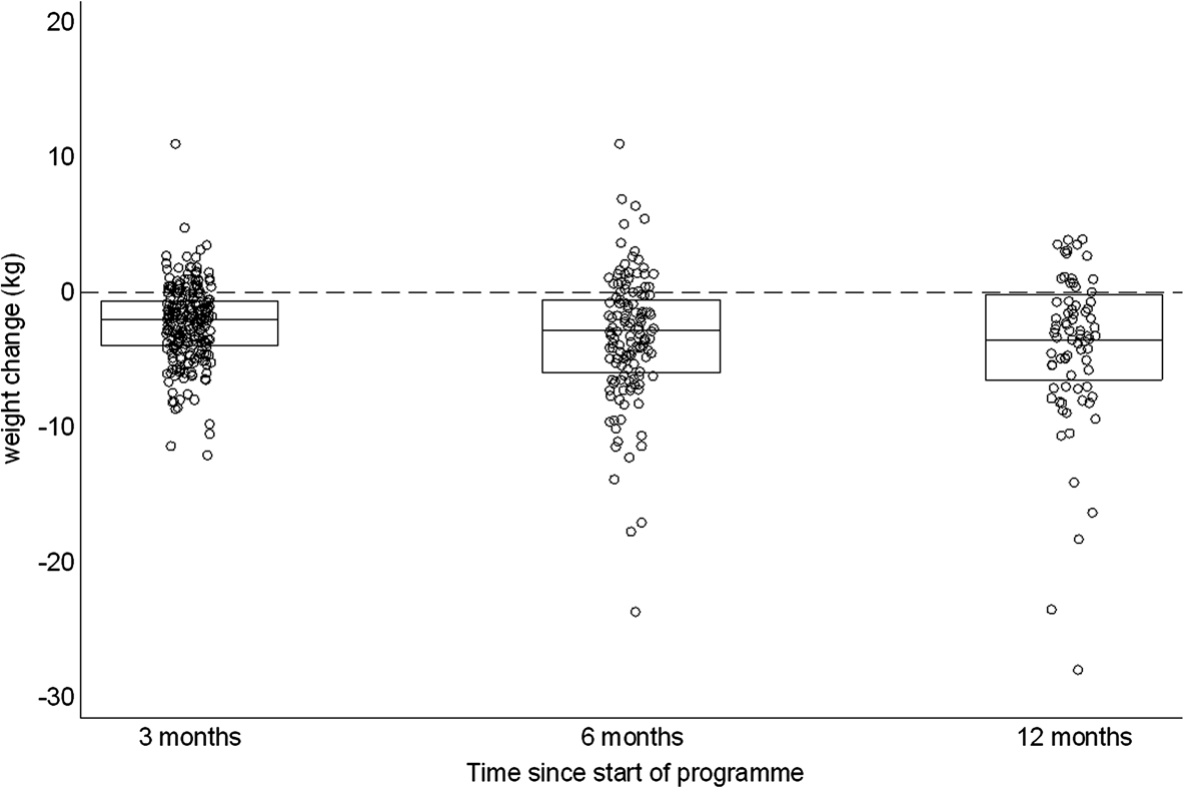
**Dot plot comparing weight change at 3, 6, and 12 months since enrollment.** Boxes indicate 25th, 50th and 75th percentiles.

The distribution of weight change at 3, 6 and 12 months is illustrated in Figure 1. Weight change was approximately symmetrically distributed around the mean at 3 and 6 months. At 12 months there was stronger evidence of skew in the distribution; for example 2 patients lost more than 20 kgs. There were progressively larger mean weight losses over time, from 2.4 kg at 3 months to 4.1 kg at 12 months. At 12 months, 57 patients (74% of patients who attended, 18% of all patients) had lost some weight, 15 patients (19% of patients who attended, 5% of all patients) had gained weight and 5 (6% of patients who attended, 2% of all patients) had no appreciable change in weight since baseline (absolute change ≤ 250 g). The maximum weight loss was 27 kg and the maximum weight gain 4.6 kg at 12 months. Weight change, expressed as a percentage of baseline weight, was similar to absolute weight change because the mean baseline weight was close to 100 kg (data not shown).

A higher percentage of men than women attended at 12 months (Table 3). Attendance increased with age and decreased with increasing BMI but these trends were not statistically significant. Men appeared to lose more weight than women (5.8 kg vs. 3.4 kg; p = 0.66 - Table 3) but there was no difference in clinically significant weight loss (39% vs. 43%; p = 0.78). A relationship between weight loss and age or BMI was not apparent. Table 3 shows that patients aged 40-49 lost more weight than other age groups while those with a BMI 30-34 kg/m^2^ lost the least amount of weight. Statistically significant differences were not found when weight loss was modelled by sex, age and BMI individually (sex p = 0.66; age p = 0.66; BMI p = 0.21 - Table 3) or in combination. The percentage achieving ≥5% weight loss similarly did not show statistically significant associations with sex (p = 0.78), age (p = 0.86) or BMI (p = 0.86). Table 3 shows that the imputed measures of mean weight loss (BOCF and LOCF) produced substantially lower estimates of mean weight loss than estimates based only on patients who attended. Both BOCF and LOFC estimates similarly showed non-significant patterns by age and BMI.

**Table 3:** Weight change and percent of patients achieving >5% weight loss at 12 months by sex, age and Body Mass Index

	**Number of patients**	Percentage (95% CI*) of patients who attended	Mean (95% CI*) weight loss (kg)			Percentage (95% CI*) of patients losing ≥5% of baseline weight		
			**Attending patients**	BOCF**	LOCF***	**Attending patients**	BOCF**	LOCF***
**Sex**								
Men	79	29.1 (19.4, 40.4)	5.79 (2.47, 9.10)	1.69 (0.58, 2.79)	2.53 (1.41, 3.56)	39.1 (19.7, 61.5)	9.8 (6.3, 14.4)	17.7 (10.0, 27.9)
Women	234	23.1 (1.78, 29.0)	3.41 (2.25, 4.57)	0.79 (0.47, 1.11)	1.46 (1.05, 1.86)	42.6 (29.2, 56.8)	11.4 (5.3, 20.5)	15.4 (11.0, 20.7)
*p value*		*0.28*	*0.66*	*0.62*	*0.21*	*0.78*	*0.69*	*0.62*
**Age group (years)**								
40-49	61	19.7 (10.6, 31.8)	4.78 (1.50, 8.06)	0.94 (0.16, 1.72)	1.49 (0.56, 2.43)	50.0 (21.1, 78.9)	9.8 (3.7, 20.2)	16.4 (8.2, 28.1)
50-59	134	24.6 (17.6, 32.8)	3.50 (1.40, 5.59)	0.86 (0.30, 1.43)	1.66 (1.01, 2.32)	39.4 (22.9, 57.9)	9.7 (5.3, 16.0)	15.7 (10.0, 23.0)
60+	111	27.9 (19.8, 37.2)	4.29 (2.36, 6.22)	1.20 (0.56, 1.84)	1.91 (1.22, 2.61)	38.7 (21.8, 57.8)	10.8 (5.7, 18.1)	16.2 (9.9, 24.4)
*p value*		*0.49*	*0.66*	*0.77*	*0.57*	*0.78*	*0.96*	*0.99*
**BMI grouping (kg/m**^**2**^**)**								
<30	30	26.7 (12.3, 45.9)	5.37 (2.35, 8.38)	1.43 (0.24, 2.63)	2.02 (0.78, 3.25)	75.0 (34.9, 96.8)	20.0 (7.7, 38.6)	23.3 (9.9, 42.3)
30- < 35	136	25.7 (18.6, 33.9)	2.61 (1.35, 3.88)	0.67 (0.30, 1.05)	1.40 (0.89, 1.91)	37.1 (21.5, 55.1)	9.6 (5.2, 15.8)	16.2 (10.4, 23.5)
35- < 40	80	23.8 (14.9, 34.6)	3.82 (1.09, 6.55)	0.91 (0.17, 1.64)	1.66 (0.84, 2.48)	31.6 (12.6, 56.6)	7.5 (2.8, 15.6)	13.8 (7.1, 23.3)
40+	64	23.4 (13.8, 35.7)	7.35 (3.08, 11.63)	1.72 (0.47, 2.98)	2.39 (1.08, 3.70)	46.7 (21.3, 73.4)	10.9 (4.5, 21.2)	15.6 (7.8, 26.9)
*p value*		*0.97*	*0.21*	*0.74*	*0.91*	*0.18*	*0.28*	*0.68*

*95% confidence interval. **BOFC - baseline observation carried forward. ***LOFC - last observation carried forward.

Patients who smoked had similar weight loss as patients who did not smoke (Table 4). Patients with diabetes who attended at 12 months appeared to lose less weight compared to patients without diabetes (1.8 kg vs. 4.6 kg; p = 0.24 - Table 4), but a difference was not apparent when the proportions (LOCF) with clinically significant weight loss were compared (15% vs. 17%; p = 0.78). Patients who did not report smoking or diabetes status appeared to be less likely to attend at 12 months (p = 0.07 & p = 0.08 respectively - Table 4).

**Table 4:** Weight change and percent of patients achieving >5% weight loss at 12 months by smoking and diabetes status

		**Number of patients**	**% attending**	Mean 12 month weight loss (kg) (95% CI*)	% losing 5% weight (BOCF**) (95% CI*)	% losing 5% weight (LOCF***) (95% CI*)
**Smoking status**	smokers	45	28.9	4.47 (0.60, 8.35)	11 (4, 24)	13 (5, 27)
	non smokers	210	26.2	4.19 (2.60, 5.79)	11 (7, 16)	17 (12, 22)
	not recorded	59	15.3	3.16 (0.69, 5.63)	7 (2, 16)	15 (7, 27)
	*p value*		*0.17*	*0.99*	*0.63*	*0.85*
**Diabetes status**	diabetes	33	21.2	1.78 (-0.80, 4.34)	3 (0, 16)	15 (5, 32)
	no diabetes	228	27.2	4.57 (3.02, 6.13)	12 (8, 17)	17 (12, 23)
	not recorded	53	15.1	2.66 (0.12, 5.12)	6 (1, 16)	11 (4, 23)
	*p value*		*0.16*	*0.36*	*0.13*	*0.58*

*95% confidence interval.

**BOCF - baseline observation carried forward.

***LOFC - last observation carried forward.

## Discussion

Our study reports on the largest prospective evaluation of a pharmacy based weight management programme in the UK over a twelve month follow-up period. We found that 10% of patients enrolled in a community pharmacy-based weight management programme had lost ≥5% of baseline weight after 12 months. Measurement of successful weight loss maintenance at 12 months requires both initial weight loss and continued attendance and we found that these did not differ significantly by age, sex or baseline BMI. Attendance was highest among men, and appeared to increase with age and decrease with increasing BMI. Weight loss – measurable only among attendees – was greatest in men and patients under 50 but showed no clear relationship to BMI.

Jolly’s randomised controlled trial, Lighten Up, reported mean weight losses of 1.19 kg (95% CI -0.7 to 3.1), corresponding to 14.3% of the cohort (using BOCF) losing ≥5% at one year among users of a pharmacy-based weight management programme [12]. Weight losses in our pharmacy trial were similar, with mean loss at 12 months of 1.01 kg (0.64, 1.38) (BOCF) but the proportion achieving a ≥5% loss was lower, at 10.2%. Lighten Up pharmacy patients had lower baseline BMIs than our pharmacy group (96% vs. 78% respectively with BMI < 40), they were younger (mean age 49 vs. 54 years) but a similar proportion were men (27% vs. 25%). As we did not find significant effects of either BMI or age on weight loss, it seems unlikely that these explain the observed difference in outcomes. However, 20% of weights reported in the Lighten Up pharmacy group were self-reported and weights were available for 57% of patients, compared to our 25%. It would seem that the community pharmacies delivering the Counterweight Programme were poorer at retaining patients but more effective in achieving clinically meaningful weight losses among those who attended. Assessment of readiness to change, for example the stages of change model by Prochaska [20], is widely used in health promotion interventions and is part of the Counterweight Programme. Such information was not collected as part of routine data recording in our study. Improved screening with regular review of motivation may improve retention and efficacy of the programme. Among the three pharmacy studies identified by Gordon and others that reported 12-month outcomes [13], one reported mean weight losses of up to 2.4 kg (2.7%) with the addition of high risk counselling [21]; another reported 1.9 kg mean weight loss [22]; and a third reported mean weight loss of 4.1 kg [23,24]. Toubro and others’ study [23], however, used baseline and subsequent self-reported weights only, and is possibly affected by reporting bias.

Evidence-based weight loss programmes for adults in the UK report 12 month mean weight loss ranging from 1.1 to 6.6 kg, and they achieve 5% weight loss for between 14% to 46% of patients [25]. Commercial community-based organizations such as Weight-Watchers appear to be more effective [26], but a direct comparisons between programmes is difficult because of differences in the case-mix of patients they serve and the context in which the programmes are delivered. All programmes suffer from high attrition which limits the ability to compare outcome data. Effective ways to increase retention and attendance are needed, and this may improve weight loss outcomes by increasing the time spent in programme participation. Weight loss programmes based within community pharmacies have the attraction of being widely accessible which may increase participation. Our study, however, found that when the Counterweight Programme was based in pharmacies, attendance at 12 months was comparable to that achieved when the programme was delivered in general practice (25% vs. 28%) [27].

Our study has several strengths and weaknesses. Its strengths are that it reports weight loss using a clinically-based threshold of ≥5% rather than a mean weight change in a patient group; weights were objectively measured and not self-reported; and that it describes long-term weight loss rather than end-of-programme results. Weaknesses of the study include possible unrepresentativeness of the patients or pharmacies and a lack of detailed information about other social and clinical factors that may have influenced patients’ attendance and weight loss. The Keep Well population, for example, is composed of patients from disadvantaged areas, identified at being at high risk of ill health, and who are not fully engaged with primary care services. The present study was not an RCT and we did not employ a comparison group. It is difficult to recruit (particularly to a control group) and conduct such a study when pharmacies were the main delivery point of the weight management programme in localities where the study population was composed mainly of patients from disadvantaged backgrounds.

In established practice the Counterweight Programme delivered in primary care achieved ≥5% weight loss at 12 months in 10% of patients [27] and this Counterweight Programme delivered in pharmacies also achieved the same weight loss in 10% of patients. Our study indicates the effectiveness of a programme delivered in areas where GPs would not provide Counterweight services and we therefore suggest that pharmacy-delivered weight management remains an option that should be considered where alternatives are not available.

## Conclusions

In conclusion, we have demonstrated that a pharmacy-based weight management programme achieves clinically significant, objectively-measured weight losses at 12 months in 10% of patients who enrol. There are few other evaluations of long-term weight loss outcomes in community pharmacies and several include self-reported weights, which may be subject to significant reporting biases. The Counterweight Programme delivered in pharmacies should be considered as part of a range of services available to a community to manage overweight and obesity.

## Competing interests

LM and NB are employees and shareholders of Counterweight Ltd. The other authors have declared no competing interests.

## Authors' contributions

DM & PM were responsible for the statistical analyses and drafting and writing the manuscript. AS, JG, LM and NB arranged and coordinated pharmacy involvement, data acquisition and contributed to the drafting of the paper. All authors read and approved the final manuscript.

## Pre-publication history

The pre-publication history for this paper can be accessed here:

http://www.biomedcentral.com/1471-2458/13/282/prepub
